# A Dual-Attention Recurrent Neural Network Method for Deep Cone Thickener Underflow Concentration Prediction

**DOI:** 10.3390/s20051260

**Published:** 2020-02-26

**Authors:** Zhaolin Yuan, Jinlong Hu, Di Wu, Xiaojuan Ban

**Affiliations:** 1 School of Computer and Communication Engineering, University of Science & Technology Beijing, Beijing 100083, China; b20170324@xs.ustb.edu.cn (Z.Y.); g20188793@xs.ustb.edu.cn (J.H.); 2 Department of ICT and Natural Science, Norwegian University of Science and Technology, 6009 Ålesund, Norway; di.wu@ntnu.no

**Keywords:** time series prediction, dual-attention, spatio-temporal relationship, cone thickener, industrial internet of things (IIoT)

## Abstract

This paper focuses on the time series prediction problem for underflow concentration of deep cone thickener. It is commonly used in the industrial sedimentation process. In this paper, we introduce a dual attention neural network method to model both spatial and temporal features of the data collected from multiple sensors in the thickener to predict underflow concentration. The concentration is the key factor for future mining process. This model includes encoder and decoder. Their function is to capture spatial and temporal importance separately from input data, and output more accurate prediction. We also consider the domain knowledge in modeling process. Several supplementary constructed features are examined to enhance the final prediction accuracy in addition to the raw data from sensors. To test the feasibility and efficiency of this method, we select an industrial case based on Industrial Internet of Things (IIoT). This Tailings Thickener is from FLSmidth with multiple sensors. The comparative results support this method has favorable prediction accuracy, which is more than 10% lower than other time series prediction models in some common error indices. We also try to interpret our method with additional ablation experiments for different features and attention mechanisms. By employing mean absolute error index to evaluate the models, experimental result reports that enhanced features and dual-attention modules reduce error of fitting ~5% and ~11%, respectively.

## 1. Introduction

**Figure 1:**
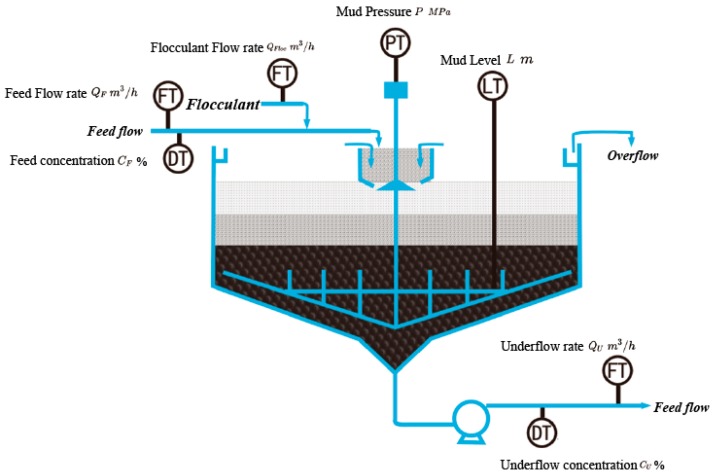
Working process of thickener is continuous. Crude low concentration slurry flow was fed into the mix tank accompanied with flocculant. The dissolved particles agglomerate to larger lump under the effect of flocculant and concentrate at the bottom of thickener. Underflow with high concentration is produced and clear water will be recycled from the overflow pipe which locates at the top of thickener.

Deep cone thickener, also named paste thickener, is an important equipment in industrial mining process, especially for sustainable mining environment protection. It is a giant complex system to generate raw material for backfill paste in the processed mines. A general framework of thickener and key processing parameters are illustrated in Figure 1.

Stable underflow concentration is a fundamental index to discriminate against the performance and stability of industrial production process. Many parameters during production affect the stability of underflow concentration. Unstable volume and concentration of feed flow disturb the mass balance of mud bed in thickener. This usually leads to underflow concentration oscillation. Other parameters, such as flocculant dosage and underflow volume, also affect the underflow concentration. In industrial thickener production process, underflow concentration prediction is the top priority for further system control.

The current thickener system is highly depending on massive integrated sensors to monitor and control the production process, known as thickener with Industrial Internet of Things (IIoT) [1]. From this system, data are collected on real time from all the sensors and provide decision support for operators and managers [2]. These data are also useful for future equipment diagnosis.

Traditional underflow concentration can be modeled as a typical multidimensional time series prediction formulation. The change of underflow concentration obeys an unknown distribution in time domain which can be formulated by $p(y_{t+1}-y_{t}|y_{1},\ldots ,y_{t-1},y_{t})$ with $y_{t}\in R$. Expect for underflow concentration, some other relevant series, which are monitored from different sensors, provide additional prior knowledge to predict underflow concentration in future. Formally, we assume *n* additional sensors are considered and all sensors capture the processing values at the same time. $x_{t}\in R^{n}$ represents a group of monitored values from *n* sensors at time step *t*. Theoretically, distribution $p(y_{t+1}-y_{t}|y_{1},\ldots ,y_{t-1},y_{t},x_{1},\ldots ,x_{t-1},x_{t})$ has lower entropy than $p(y_{t+1}-y_{t}|y_{1},\ldots ,y_{t-1},y_{t})$. This paper focuses on the construction of such a multidimensional time series prediction model, which can predict $y_{t+1}$ according to previous seen spatial features $\left(x_{1},\ldots ,x_{t-1},x_{t}\right)$ and temporal features $\left(y_{1},\ldots ,y_{t-1},y_{t}\right)$. Most of these studies modeled the thickener system mostly with mathematical methods [3,4] or data-driven methods [5]. Mathematical models give state equations of underflow concentration. These equations are deduced from the physical and structural law. However, these methods suffered from the complexity of thickener system and external environment disturbance. Therefore, they are restricted for accuracy and universality. Data-driven system identification has better adaptability and better performance than conventional mathematical model-based methods [6,7]. In this paper, for problem setting, we have collected massive sensor data from the concrete industrial process. After the discussion with the domain expert, the aim is to build relationship between sensor data and underflow concentration values. For that reason, we need an end-to-end regression model based on sufficient training data.

Conventional time series prediction models are widely used in industrial analysis, such as autoregressive integrated (AR) [8], autoregressive integrated moving average (ARMA) [9], recurrent neural network and Long Short-Term Memory (LSTM) [10]. These methods achieved much success in various industrial fields. Here, we list two main challenges in cone thickener systems:

- Long time delay. It occurs inevitably during the change of underflow concentration. In practice, one parameter evolves and can affect the concentration after a long time interval. In addition, the influence levels can vary over time.
- Unknown spatial sensor correlations. Different parameters in the system can affect the underflow concentration in distinct and complex forms. The challenge is that these complex interactions are still unknown from domain knowledge.

To overcome these challenges, we seek a model which can both encode the long time series and explore useful features from high-dimensional and plenty of data adaptively. Therefore, in this paper, we propose a dual-attention recurrent neural network method to solve this question. It generally includes two mechanisms: encoder and decoder. They are used to capture the spatial and temporal features from original sensor data and predict underflow concentration accurately in the thickener. To further enhance the accuracy of model, we also introduce some domain knowledge of the thickener system into the design of model. The numerical relationships between concentration, density, volume and mass are considered in our feature designing. Our industrial case study results show that the dual-attention mechanisms and added features play an important role in this problem. In addition, this method outperform the other commonly used time series predict models in comparative accuracy and efficiency.

The contributions of our work are listed as follows.

- We propose a dual-attention time series prediction model to predict the underflow concentration in the thickener system. It consists of encoder and decoder. The encoder is used to capture spatial importance of the inputted high-dimensional series. The decoder is used to capture temporal importance of the inputted long time series.
- Feature enhancement are designed based on domain knowledge for underflow concentration prediction.
- This method is applied in a concrete case study with Tailings Thickner from Metso. The data are collected directly from the industrial mining process. The prediction results show this method outperforms both in accuracy and efficiency.

The remaining part of the paper is organized as follows. Section 2 reviews the related studies about thickener system identification, data-driven data analysis methods, and attention-recurrent neural network. Section 3 introduce the details of proposed method, including basic formulation, feature enhancement methods, and model structure. Section 4 presents extensive experiments to evaluate the proposed methods and verify the effectiveness of model details. Section 6 gives the conclusion and discusses the meaningful future work directions.

## 2. Related Work

The thickening of tailing slurry is the primary process of paste filling. It is a critical procedure in modernized mining [11]. In thickening process, too high concentration can lead to accidents such as pipe plugging. In the opposite side, too low concentration will decrease the strength of backfilled paste and further reduce safety level of the whole mining process. Therefore, it is significant to predict the change of underflow concentration for the operators to keep concentration stable. Underflow concentration prediction can be seen as a system identification field based on the thickener itself with complex physical process inside. Here, we discuss two general research categories: model-based simulation and data-driven system identification.

### 2.1. Model-Based Thickener System Simulation

One typical solution is to build a mathematical function for system input and underflow concentration to predict the dynamic thickening process. This function is usually with the form of differential equations. Based on this model, the future underflow concentration can be calculated directly or by numerical integration method. A thickener dynamic model based on the sedimentation consolidation theory is proposed in [4,12]. The authors of [3] extend a one-dimensional model for the dynamics of a flocculated suspension in a clarifier-thickener to include the discharge yield stress and particle size distribution in a manner that is computationally tractable.

Mathematical methods can be explained and accurate dynamical equation could be helpful for other works, such as fault detection and optimal control. It usually suffered from the complexity of slurry particles dynamics and external unknown environment disturbance. Most dynamical models are built on lots of ideal hypotheses, which cannot often be satisfied in practical industrial process.

### 2.2. Data-Driven Thickener System Identification

In contrast, another idea which is widely used in the current IIoT systems. Ref. [13,14,15,16] adopted the data-driven method for learning a parameterized model from the real system trajectories. This method lessens the difficulty of theoretical analysis and learns from data directly. Normally, learned parameterized model performs better than conventional purely model-based method on a specific dataset. In The Internet of Things(IoT), Xiao et al. [5] analyzed the characteristics of the thicker washing process and propose the hybrid model combining mechanism modeling and error compensation model based on Extreme Learning Machine algorithm [17]. The results show that the prediction error of the hybrid model is lower than that of the mechanism model. Zhang et al. [18] designed a deep neural network model to predict equipment running data and improve the accuracy by systematic feature engineering and optimal hyper parameter searching.

Inspired by some theories of human attention [19], an encoder–decoder with attention recurrent neural network has been used in industrial systems [20]. Attention mechanisms can capture the long-term temporal dependencies appropriately and select the relevant feature series to assist the prediction module. In this work, we follow the basic structure of encoder–decoder model to construct our recurrent neural network.

From the perspective of data, feature enhancement is a key process of feature engineering in machine learning tasks [21]. The trained model can performs much better by learning from sophisticated features. In this paper, we will also build several additional features according to the prior knowledge of thickening system.

### 2.3. Summary

Table 1 compares the detailed properties contributions of each reference and the proposed method. It suggests that the proposed DARNN method has better accuracy with the benefit from the design of network structure and input features. However, the pure deep neural network framework makes the model have less interpretability and it is hard to transfer the model from one thickener to another.

## 3. Methods

**Figure 2:**
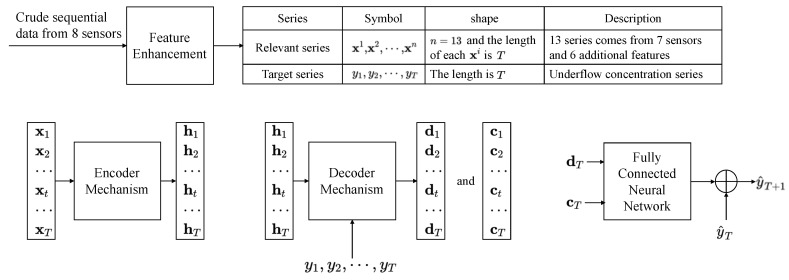
The proposed model is mainly composed four parts: Feature Enhancement, Encoder mechanism, Decoder mechanism, and a normal neural network for predicting. Feature Enhancement explores additional 6 features based on industrial experience. The left three learnable parts are connected in a chain to give the prediction and train together.

This section will first introduces the mathematical formulation of solved problem and shows the model details from two aspects: Feature enhancement and Dual-Attention mechanism for high-dimensional time series prediction. The overall illustration of the proposed method is shown in Figure 2.

### 3.1. Problem Formulation and Variable Definition

The underflow concentration prediction problem belongs to time series analysis field. *n* sensors installed in thickener monitor parameters $x_{t}={\left[x_{t}^{1},x_{t}^{2}\ldots ,x_{t}^{n}\right]}^{T}$ and underflow concentration $y_{t}$ by physical signal transmitter module. Details of state parameters *x* are shown in Table 2. All of employed monitoring points are designed from industrial perspective and have direct or indirect impact to the change of underflow concentration in future. The statistical relationships among various sensors installed in separate positions are named spatial relationships. The statistical relevance of sensors in the time dimension are named temporal relationship. Two kinds of relationships are employed in the proposed model to predict the future underflow concentration.

Collected data will be stored in historical database which is usually installed in Distributed Control System (DCS) system. To predict the future unknown underflow concentration, historical data $\left(x_{t-T+1},\ldots x_{t-1},x_{t}\right)$ and $\left(y_{t-T+1},y_{t-1},y_{t}\right)$ are exploited to estimate ${\hat{y}}_{t+1}\in R$. Our goal is to make ${\hat{y}}_{t+1}$ closed to $y_{t+1}$. The question above can be summarized as a minimization problem shown in (1).

(1) $$\min_{f}\left(E{\left({\hat{y}}_{t+1}-y_{t+1}\right)}^{2}\right),{\hat{y}}_{t+1}=f\left(x_{t-T+1},\ldots x_{t-1},x_{t},y_{t-T+1},\ldots ,y_{t-1},y_{t}\right)$$

An optimal model *f* is desired to minimize the mean square error between estimated ${\hat{y}}_{t+1}$ and real $y_{t+1}$ over the probability distribution of input which are assigned by thickener system.

### 3.2. Feature Enhancement

Many researchers demonstrate that solid mass of the mud bed, m(t), makes a strong impact to underflow concentration. Meanwhile, based on mass balance law, changes of the total solid mass of mud bed mainly depend on the solid mass flow of feeding and discharging changes [22]. Therefore, the changed solid mass can be calculated by (2).

(2) $$\begin{array}{cc} \frac{dm(t)}{dt} & =v\left(t\right)=Q_{F}\left(t\right)C_{F}\left(t\right)\phi_{F}\left(t\right)-Q_{U}\left(t\right)C_{U}\left(t\right)\phi_{U}\left(t\right)\\ m(t) & =m\left(t-1\right)+\int_{t-1}^{t}v\left(t\right)dx \end{array}$$

We assume the flow speed and concentration change linearly and let *I* is the data sampling interval. Therefore, the current solid mass in tank can be simplified to (3),

(3) $$m\left(t\right)=m\left(t-1\right)+\left(v\left(t\right)+v\left(t-1\right)\right)\times \frac{I}{2}$$

where $\phi_{U}\left(t\right)$ and $\phi_{F}\left(t\right)$ are the real-time density of underflow and feed flow, respectively. The relationship of density and concentration for tailing slurry usually obeys the quadratic function in (4):

(4) $$\phi_{U}=aC_{U}^{2}+b*C_{U}+c$$

We adopt physical detection methods to measure the concentration and density data from plenty of slurry samples. The parameters in the equation are fitted and the result is: a=1.2198, b=0.2390, c=1.0510.

Finally, we add six additional features to represent the properties of solid mass in Table 3:

To the end of the paper, the features for prediction we utilize are $x_{t}={\left[Q_{F}\left(t\right),C_{F}\left(t\right),P\left(t\right),Q_{Floc}\left(t\right),R_{s}\left(t\right),L\left(t\right),Q_{U}\left(t\right),\phi_{F}\left(t\right),\phi_{U}\left(t\right),m_{in}\left(t\right),m_{out}\left(t\right),v\left(t\right),m\left(t\right)\right]}^{T}$ and $y\left(t\right)=C_{U}\left(t\right)$.

### 3.3. Dual-Stage Attention-Based Mechanism for High-Dimensional Time Series Prediction

This paper employs a time series prediction model named DARNN for predicting underflow concentration. In the subsection, the structure of DARNN will be introduced at first and then we will explain how to model underflow concentratioin prediction problem based on DARNN model.

To simplify the expression in this part, we make a little change on the input series. For the given input sequence $X=(x_{t-T+1},\ldots x_{t-1},x_{t})$ and $y=(y_{t-T+1},\ldots y_{t-1},y_{t})$, we rewrite the indexes of each series to construct equivalent $X=(x_{1},\ldots x_{T-1},x_{T})$ and $y=(y_{1},\ldots y_{T-1},y_{T})$. Correspondingly, our goal is changed to estimate the ${\hat{y}}_{T+1}$ as accurate as possible.

#### 3.3.1. The Relationship Between DARNN and RNNs Family

RNNs are a family of architectures that have been used to model squential problems, as their hidden states carry information of past input series. As one of the most popular architecture, the encoder–decoder framework parts the sequence translation process into two phases and it is widely used in machine translation and sequence generation. Two stacked RNNS build the architecture. The first one is named encoder, which encodes the input series of arbitrary dimension to a vector representation in a fixed-length space. The second RNN is named decoder, which decodes the vector representation above to a target sequence. Two modules are trained together to minimize the loss penalty of the output target sequence. The two processes above can be formulated as $f_{1}$ and $f_{2}$:

(5) $$\mathrm{Encoding}\;\mathrm{stage}:\;h_{t}=f_{1}\left(x_{t},h_{t-1}\right)$$

(6) $$\mathrm{Decoding}\;\mathrm{stage}:\;d_{t}=f_{2}\left(h_{t},d_{t-1}\right)$$

Some references [23,24] show that when the dimentions of input sequence increase, fixed-length representation cannot encode the high-dimensional sequence well, which makes the performance dropped rapidly. To confront this problem, a mechanism named attention is employed in decoding stage which assign the weights of hidden states $h_{j}$ dynamically at each time step. The formulation of decode stage is changed to (7):

(7) $$\mathrm{Decoding}\;\mathrm{stage}:\;d_{t}=f_{2}\left(c_{t},d_{t-1}\right)$$

with (8):

(8) $$c_{t}=\sum_{i=1}^{T}\beta_{t}^{i}h_{i}$$

The attention weight $\beta_{t}^{i}$ represents the temporal importance of encoded information. It is calculated by (9) and (10):

(9) $$l_{t}^{i}=v_{d}^{⊤}\tanh\left(W_{d}\left[d_{t-1};h_{i}\right]\right),\;1\leq i\leq T$$

and

(10) $$\beta_{t}^{i}=\frac{\exp\left(l_{t}^{i}\right)}{\sum_{j=1}^{T}\exp\left(l_{t}^{j}\right)}$$

where $\left[d_{t-1};h_{i}\right]\in R^{p+m}$ is a concatenation of previous hidden state in decoding stage and the output from encoder mechanism. $v_{d}\in R^{m}$ and $W_{d}\in R^{m\times (p+m)}$ are parameters to learn. The fully connected neural network determined by parameters $\left(v_{d},W_{d}\right)$ is shared to each $h_{i},1\leq i\leq T$. Decoder predicts the target sequence conditioned on time-varing hidden vector $c_{t}$. Plenty of successes in sequence modeling tasks make the encoder–decoder framework used in almost all advanced recurrent architectures.

Some theories of human attention [19] argue that behavioral results are best modeled by a two-stage attention mechanism. Human attention system can select elementary stimulus features in the early stages of processing. Based on the encoder–decoder framework, a new network structure, named dual-stage attention-based recurrent neural network (DARNN) is proposed in [25]. Compared with single attention encoder–decoder architecture, DARNN adds the consideration about the weighted-importance of input relevant series. In the encoding stage, an input attention mechanism is used to adaptively select the importance for every component $x_{t}^{k}$ at each time step *t*. The encoding process (5) is updated to (11):

(11) $$\mathrm{Encode}\;\mathrm{stage}:\;h_{t}=f_{1}\left({\tilde{x}}_{t},h_{t-1}\right)$$

Each original component is transformed to a weighted one with (12):

(12) $${\tilde{x}}_{t}={\left(\alpha_{t}^{1}x_{t}^{1},\alpha_{t}^{2}x_{t}^{2},\ldots ,\alpha_{t}^{n}x_{t}^{n}\right)}^{⊤}$$

Attention weight $a_{t}^{k}$ is determined by hidden state $h_{t-1}$ and the complete *k*th relevant sequence $x^{k}=\left[x_{1}^{k},x_{2}^{k},\ldots ,x_{T}^{k}\right]$ in all time steps. Here, another fully connected network and a softmax normalization are employed in the second attention model:

(13) $$e_{t}^{k}=v_{e}^{⊤}\tanh\left(W_{e}\left[h_{t-1};x^{k}\right]\right),\;1\leq k\leq n$$

and

(14) $$\alpha_{t}^{k}=\frac{\exp\left(e_{t}^{k}\right)}{\sum_{i=1}^{n}\exp\left(e_{t}^{i}\right)}$$

where $h_{t-1}$ is hidden state of encoder, and $v_{e}\in R^{T}$ and $W_{e}\in R^{T\times (m+T)}$ are learnable parameters and shared to each relevant sequence $x^{k}$. With the above attention mechanism, $h_{t}$ carries the deeply encoded information of $x_{t}$ accompanied with the input information from other time step $x_{i}$ where i≠t.

#### 3.3.2. Modelling Underflow Concentratioin Prediction Problem based on DARNN Model

**Figure 3:**
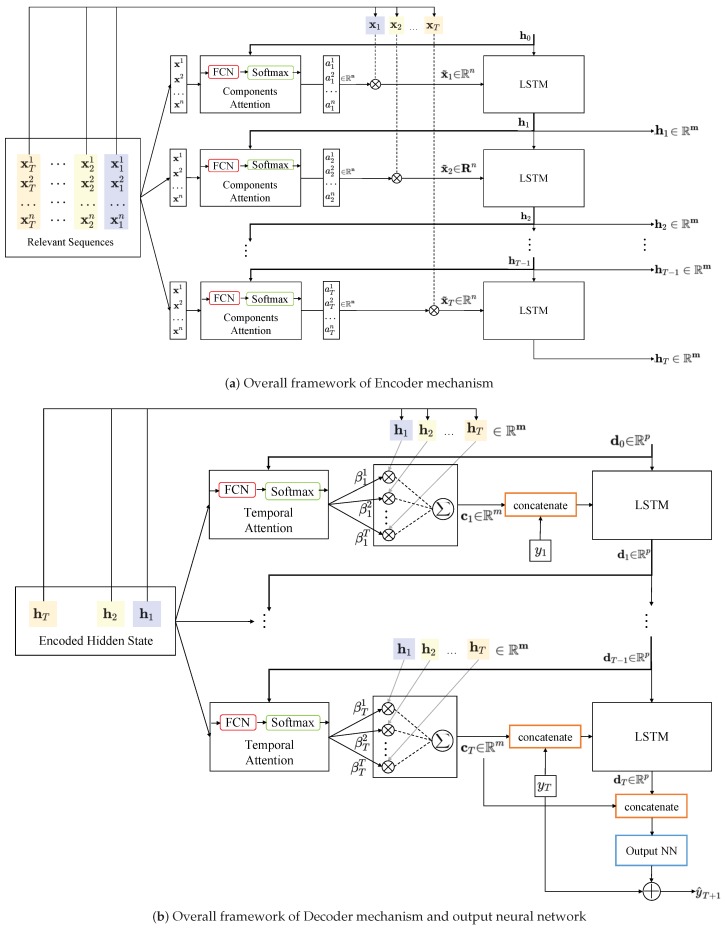
The proposed model consists of three parts: encoder, decoder, and a fully connected neural network for final predicting. The output of the encoder mechanism is the input of the decoder mechanism. Encoder is employed to embed the history series to encoded features $h_{t}$, which are inferred from a Lstm mechanism in encoder module. Then, the encoded features will be decoded by decoder module and produce new hidden state $d_{t}$. The third neural network estimates the difference between $y_{t+1}$ and $y_{t}$ from $d_{t}$ and another context features $c_{t}$. (**a**) Overall framework of Encoder mechanism; (**b**) Overall framework of Decoder mechanism and output neural network

This paper follows the concept of DARNN framework and solves the high-dimensional underflow concentration prediction problem with a Temporal and Spatial Attention Mechanism. A graphical illustration of the model is shown in Figure 3.

As Figure 2 shows, the complete model is a learnable chain that consists of three main parts: encoder, decoder, and a global residual network for predicting the underflow concentration. The work flow of encoder and decoder has been introduced in the last part. There is a slight difference in proposed method that the underflow concentration sequence. $y=(y_{1},\ldots y_{T-1},y_{T})$ is not encoded by the encoder mechanism. Because the sequence y is a shallow feature, which has straightforward statistic relationship with predicted ${\hat{y}}_{T+1}$, it does not need to encode the y like other relevant series X. We make it as a part of the input of decoder mechanism. Therefore, the equation of decoding process (7) is changed to (15).

(15) $$\mathrm{Decoding}\;\mathrm{stage}:\;d_{t}=f_{2}\left(c_{t},y_{t},d_{t-1}\right)$$

$f_{1}$ and $f_{2}$ in (15) and (11) are all LSTM unit, which is defined in (16)–(20).

(16) $$f_{t}=\sigma \left(W_{f}\left[h_{t-1};x_{t}\right]+b_{f}\right)$$

(17) $$i_{t}=\sigma \left(W_{i}\left[h_{t-1};x_{t}\right]+b_{i}\right)$$

(18) $$o_{t}=\sigma \left(W_{o}\left[h_{t-1};x_{t}\right]+b_{o}\right)$$

(19) $$s_{t}=f_{t}⊙s_{t-1}+i_{t}⊙\tanh\left(W_{s}\left[h_{t-1};x_{t}\right]+b_{s}\right)$$

(20) $$h_{t}=o_{t}⊙\tanh\left(s_{t}\right)$$

The key reason for using an LSTM unit is that it can overcome the problem of vanishing gradients and better capture long-term dependencies of time series. This advantage is especially useful for thickener system prediction because long time delay often occurs when system changes. Finally, encoder and decoder modules transform the original input sequences y and X to another high-dimensional feature sequences $\left(d_{1},d_{2},\ldots ,d_{T}\right)$ and $\left(c_{1},c_{2},\ldots ,c_{T}\right)$. Another network module reserves the feature representation in last time step *T* and produce the desired ${\hat{y}}_{T+1}$ in (21).

(21) $$\begin{array}{cc} {\hat{y}}_{T+1} & =F\left(y_{1},\ldots ,y_{T},x_{1},\ldots ,x_{T}\right)\\ & =v_{y}^{⊤}\tanh\left(W_{y}\left[d_{T};c_{T}\right]+b_{w}\right)+b_{v}+y_{T} \end{array}$$

$\left[d_{T};c_{T}\right]\in R^{p+m}$ is a concatenation of the decoder hidden state and the context vector. A single hidden layer neural network composed with learnable input layer $\left(W_{y},b_{w}\right)$ and hidden layer$\left(v_{y},b_{v}\right)$ is utilized to produce the final prediction result. The usage of $c_{T}$ in the last prediction phase could be explained from multi-level feature fusion perspective [26]. Because $c_{T}$ is the weight-sum of $\left(h_{1},h_{2},\ldots ,h_{T}\right)$, it includes all the embedded information from encoder module. This skip connection plays a similar role to maintain the range of gradient just like res-block or dense-block [27].

Furthermore, there is a bias term $y_{T}$ in the (27), which means the model does not learn the underflow concentration $y_{T+1}$, but the difference $\Delta y=y_{T+1}-y_{T}$. Because the underflow concentration almost changes in continuous way. In adjacent two time steps, underflow concentration in next time step $y_{T+1}$ is approximately equal to the current value $y_{T}$. This trick makes the model employs the prior information from $y_{T}$ more adequately. Experimental result shows that the bias term results in much lower initial model error before training than no-bias schema and the model could converge to best parameters rapidly.

#### 3.3.3. Model Training

All of operations in our model are smooth and differentiable, so we can train the model by standard back propagation algorithm with the loss function defined in (22),

(22) $$O\left(y_{t+1},{\hat{y}}_{t+1}\right)=\frac{1}{N}\sum_{i=1}^{N}\left|{\hat{y}}_{t+1}-y_{t+1}\right|$$

where *N* is the number of training samples. More details of the training will be introduced in next part. The code is implemented by pyTorch and the source code can be found in github (https://github.com/Kyrie-Hu/Thickener-Underflow-Concentration-Prediction).

## 4. Industrial Case Study

In this section, we first describe the dataset collected from the our thickener IIoT platform. Detailed experimental settings are given with comparative results against LightGBM, RNN, and LSTM on prediction accuracy. To provide explanations of this method, ablation tests are done for further analysis of the attention mechanisms.

### 4.1. IIoT Platform

**Figure 4:**
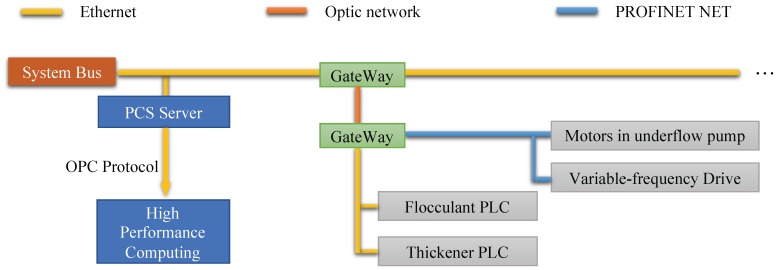
The topology graph of each devices and servers in the industrial case. We delete some components in the graph which are not related to our problem, such engineer station, operator station, etc. Historical database and prediction program are all deployed in high-performance computing server.

This study is based on an IIoT platform to support the communication among sensors, industrial equipment, distributed control system, and high-performance computing server. The topology graph of the framework is shown in Figure 4. Details of deployed sensors in factory are listed in Table 4. A sample of the dataset is shown in Table 5. This system takes the advanced SIMATIC Process Control System PCS 7 APL in our case. Training data are all real production data and collected from the IIoT platform.

### 4.2. Data Preprocessing and System Set-Up

To verify the performance of proposed method and other baselines adequately and fairly, batches of data come from different time periods are employed to train model and test model separately. We construct training data set by using production data during May to June in 2018. Test dataset is corresponding to original data which are produced in September 2019.

We make lots of data preprocessing procedures on the origin dataset which are derived from the thickener system, including removing outlier data, deleting the interval when the system is out of service, and normalizing data to make each series indicate standard normal distribution. There are ~14,800 clean data left after preprocessing, and the sampling period between two adjacent points is 2 minutes. Each data point has a total of eight parameters including the underflow concentration column. Then, according to the correlation analysis between features, we create six additional features for each record by using the method introduced in Section 3.2.

**Figure 5:**
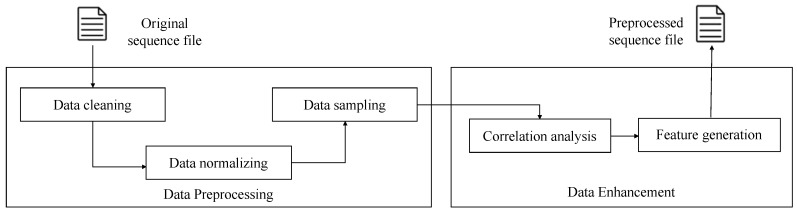
The diagram illustrating the process of data preprocessing.

Finally, we collect a dataset which has 14 features in each data point. In our study, underflow concentration is the predicted target series, and other 13 features are relevant series. The first 8847 data points from training set are used to train the model, and the following 2949 data points are the validation set which can help us find the best experimental parameters and stop the training iterations properly. Test data set has 2949 data points of all which are used as to test. A diagram illustrating the process of data preprocessing is shown in Figure 5.

We use minibatch stochastic gradient descent (SGD) together with the Adam optimizer [28]. The size of one batch is 128 and learning rate is set to 0.001 invariably.

### 4.3. Accuracy Analysis of Underflow Concentration Prediction

To demonstrate the effectiveness of our method, we compare it against three other methods. Among them, LightGBM [29] is a gradient boosting decision tree (GBDT) algorithm. It contains two novel techniques: gradient-based one-side sampling and exclusive feature bundling, dealing with the problem of large number of data instances and features, respectively. Recurrent neural network (RNN) is a classical method to address time series prediction. Long short-term memory (LSTM), which is the most popular method for time series prediction, successfully solved the problem of gradient explosion and gradient vanishing of RNN.

To measure the effectiveness of various methods for time series prediction, we consider four different evaluation metrics. Among them, root mean squared error (RMSE), root mean squared logarithmic error (RMSLE) [30], and mean absolute error (MAE) are scale-dependent measures, and mean absolute percentage error (MAPE) is a scale-independent measure. Specifically, assuming $y_{t}$ is the target at time t and $\hat{y}\;t$ is the predicted value at time t, RMSE is defined as

(23) $$RMSE=\sqrt{\frac{1}{N}\sum_{i=1}^{N}{\left(y_{t}^{i}-{\hat{y}}_{t}^{i}\right)}^{2}}$$

and MAE is denoted as

(24) $$MAE=\frac{1}{N}\sum_{i=1}^{N}|y_{t}^{i}-{\hat{y}}_{t}^{i}|$$

When comparing the prediction performance, mean absolute percentage error is popular because it measures the prediction deviation proportion in terms of the true values, i.e.,

(25) $$MAPE=\frac{1}{N}\sum_{i=1}^{N}|\frac{y_{t}^{i}-{\hat{y}}_{t}^{i}}{y_{t}^{i}}|\times 100\%$$

RMSLE is an evaluation metric from the Kaggle competition, calculated as

(26) $$RMLSE=\sqrt{\frac{1}{N}\sum_{i=1}^{N}{\left(log\left(1+{\hat{y}}_{t}^{i}\right)-log\left(1+y_{t}^{i}\right)\right)}^{2}}$$

The results of baseline methods and ours over the dataset are shown in Table 6.

In Table 6, we observe that the MAE of LightGBM is generally worse than RNN-based approaches. Because the input of LightGBM model does not include historical data points, the model cannot make full use of the historical information of sequences. For RNN-based approaches, the performance of LSTM is better than that of RNN, illustrating that LSTM is more capable to capture long-term temporal dependence which is essential in our problem.

**Figure 6:**
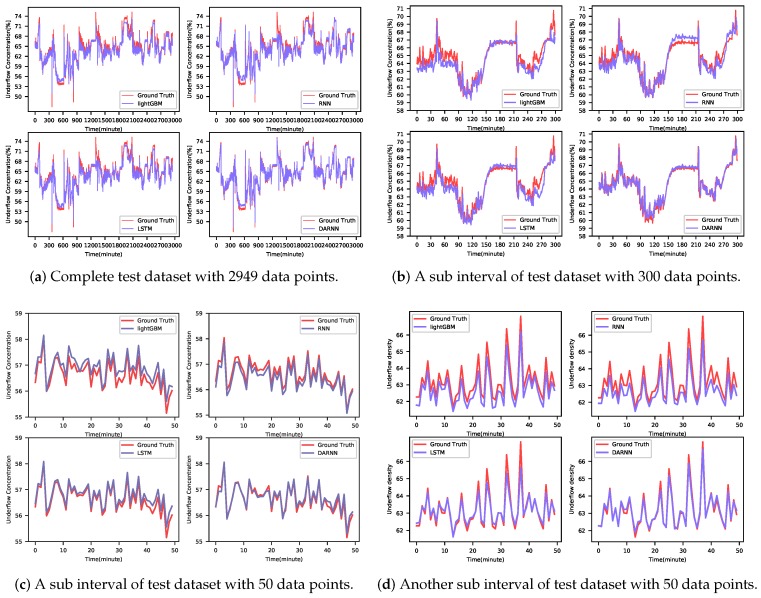
(**a**) The image shows all the data in the test set. (**b**) The image shows 300 pieces of data from the test set. (**c**,**d**): The image shows 50 pieces of data from the test set. In each image, LightGBM (upper left), RNN (upper right), and LSTM(lower left) are compared with DARNN (lower right).

DARNN method achieves the best MAE, MAPE, RMSE, and RMLSE in the dataset. It not only uses an input attention mechanism to extract relevant feature series, but also employs a temporal attention mechanism to select relevant hidden features across all time steps. Both attention mechanisms preserve meaningful features and inhibit useless features during the feedforward stage. It is a significant improvement because that attention branch makes the model no longer infer the ${\hat{y}}_{T+1}$ in statistic schema constantly. The comparison of prediction results of different algorithms is shown in Figure 6.

To further investigate the importance of input features, we designed a comparative experiment. Specifically, we generate six additional feature series through analyzing the operating characteristics of deep cone thickener. Then, we put these six enhanced feature series together with the eight original feature series as the input and test the effectiveness of our method. In Table 6, we can that clearly, using either LSTM or our method, the performance of enhanced feature series are significantly higher than that of original feature series.

### 4.4. Comparison of Temporal Attention and Spatial Attention

To verify the efficiency of two attention mechanism in our model, we make an ablation experiment to study the promotion of each attention part by deleting one or two attention modules. The experimental results are shown in Table 7.

In Table 7, the temporal attention RNN outperforms the no attention RNN. This suggests that adaptively extracting feature series can provide more reliable input features to make accurate predictions. From another aspect, the performance of spatial attention RNN are better than that of the no attention RNN. This shows that the importance of different time points in the time series can provide effective data support for the prediction. Our method combined temporal attention and spatial attention, as a result, achieving the best results in the predictions.

### 4.5. A Study on the Effect of Global Residual Connection

**Figure 7:**
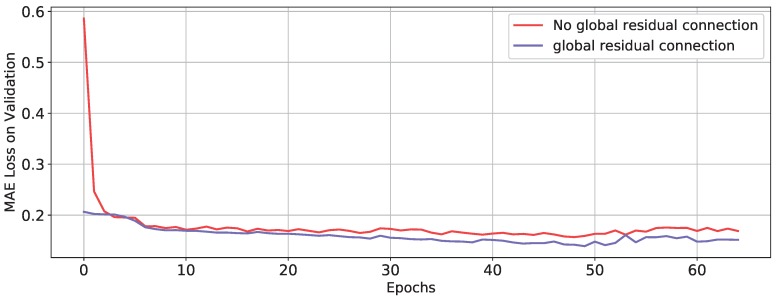
Global residual connection validation losses. The validation losses of the model which has global residual connection are slightly lower than the model does not have; at the beginning of training, the former has significant lower loss than the other.

In this subsection, an ablation experiment is conducted to study the effect of global residual connection in Equation (27). The skip connection is deleted in the compared model and two models are all trained with stochastic parameters. The validation losses of two models during training phase is illustrated in Figure 7. The improvement comes from the skip connection can be explained from the properties of thickening system. In the industrial control field, the dynamical system of thickener is always formulated as an ordinary differential equation (ODE) [3]:

(27) $$y\left(t_{1}\right)=\int_{t_{0}}^{t_{1}}h\left(y\left(t\right),x\left(t\right)\right)dt+y\left(t_{0}\right)$$

Relevant parameters x(t), such as mud pressure, feed flow rate, and the other dynamical variables and the underflow concentration y(t), make direct impact on the derivative of underflow concentration, which is defined by h(y(t),x(t)). In the proposed method, the global residual connection makes the DARNN model learn the current derivative $h(y\left(t_{0}\right),x\left(t_{0}\right))$, which can be viewed as discretizing the continuous thickening system. When $t_{0}$ is approximately equal to $t_{1}$, the difference of underflow concentration $y\left(t_{1}\right)-y\left(t_{0}\right)$ is approximately equal to the $\left(t_{1}-t_{0}\right)h\left(y\left(t_{0}\right),x\left(t_{0}\right)\right)$. In our method, the distance between two adjacent time steps is 2 minutes which is extraordinarily short for thickening process. So the error of discretization is relatively slight and prediction accuracy can be improved by simplifying the target function.

## 5. Discussion

This study supports evidence that dynamic attention branches bring into correspondence with the dynamic properties of thickener. For example, various feed concentration will not influence the underflow concentration at once. The effect will take place after a while. However, the time delay is not constant which is closely related to the height of mud bed. Many similar phenomenons exist in thickening process. Therefore, a simple sequential network without dynamic branches can hardly fit the dynamic properties well. In the perspective of the data quality, as we all know, sensors monitor industrial data by converting physical signals to electrical signals and generating the numerical values. In this process, various noises degrade the performance of sensors. In the thickener system, the prediction model not only learns to estimate the future underflow concentration, but also counteracts the noisy input and noisy feedback loss. Data with poor quality can hardly generate high quality models which perform well to predict concentration in long-time future. Compared with other models, DARNN has added parameters and a dynamical branch which improve the ability to filter the high frequency noise from the input.

Furthermore, thickening is a slow process and underflow concentration almost does not change impulsively. Compared with DARNN, other time series prediction methods all represent that the estimated underflow concentration ${\hat{y}}_{t+1}$ is extremely close to the current underflow concentration $y_{t}$. The behavior makes the model receives relatively low loss-penalty, but it has no significance for industrial demand. Because of the global residual connection, DARNN fits these tiny changes of concentration well which improves the accuracy and gives important indications to help the operator evaluate the current production and feedforward control.

## 6. Conclusions

In this paper, we present a dual-attention method for predicting the future underflow concentration of thickener system. This method also include a feature enhancement stage from domain knowledge. By considering the properties of thickener system, we produce another six derived features from original sensor data to make the model learn latent regularity of underflow concentration changes in Thickener easily. The dual-attention method is implemented by a composition of encoder and decoder mechanisms. They are used to capture both temporal information and relevant information from inputted history data.

We applied this method in an industrial IIoT platform. The results show that the enhanced features improve the prediction accuracy significantly and the proposed method outperforms other commonly used time series models. Meanwhile, two ablation experiments are conducted to prove that the contributions of different attention mechanisms and global residual connection are significant.

This method also have potential usages in other industrial time series problem which has obvious temporal and high-dimensional properties. However, numerous parameters and complex operations restrict the efficiency of the model which makes it not suitable for real-time occasion. A more lightweight network structure is expected to achieve similar performance in the future studies.

## Figures and Tables

**Table 1:** Summarizing of features and contributions of some references.

Refs	Mathematic Interpretability	Accuracy	Core Contributions
[4,12]	+++	+	Modeling complicated thickener dynamic model as a simple mathematic equation
[3]	+++	+	Add the influence of rake to the basic model
[5]	++	++	Combining mathematical thickener model and machine learning method
[20]	+	+++	Data-driven thickener modeling without human knowledge
Proposed method	+	++++	Sophisticated features design and introducing dual-attention mechanisms

**Table 2:** Detailed monitoring point list in thickener system.

Name	Symbol	Unit	Point Description
Feed flow rate	$Q_{F}$	m${}^{3}$/h	Flow speed of the feed with low concentration
Feed concentration	$C_{F}$	%	Flow concentration of the Feed with low concentration
Mud Pressure	*P*	MPa	Mud pressure at the bottom of the tank
Rake speed	$R_{s}$	rpm	Rotating speed of thickener rake
Flocculant flow rate	$Q_{Floc}$	m${}^{3}$/h	Dosage of the flocculant
Mud Level	*L*	m	Height of the slurry in the tank
Underflow rate	$Q_{U}$	m${}^{3}$/h	Flow speed of the discharged underflow
Underflow concentration	$C_{U}$	%	Concentration of the discharged underflow

**Table 3:** Detailed monitoring point list in thickener system.

Symbol	Unit	Point Description
$\phi_{F}\left(t\right)$	t/m${}^{3}$	The density of feed slurry.
$\phi_{U}\left(t\right)$	t/m${}^{3}$	The density of discharged slurry.
$m_{in}\left(t\right)=Q_{F}\left(t\right)C_{F}\left(t\right)\phi_{F}\left(t\right)$	t	The increment of solid mass from feed slurry.
$m_{out}\left(t\right)=Q_{U}\left(t\right)C_{U}\left(t\right)\phi_{U}\left(t\right)$	t	The decrease of solid mass by discharging underflow.
$v\left(t\right)=Q_{F}\left(t\right)C_{F}\left(t\right)\phi_{F}\left(t\right)-Q_{U}\left(t\right)C_{U}\left(t\right)\phi_{U}\left(t\right)$	t	The changes of solid mass in tank.
$m\left(t\right)=\sum_{i=1}^{t}=\frac{v(t)+v(t-1)}{2}\times I$	t	Cumulative changes of solid mass in tank.

**Table 4:** Details of sensors in data collection system.

Monitor Points	Detail Information of Sensors
Feed flow rate	Flow Transmitter for tailing Manufacturer: CiDra Model: SONARtrac
Feed concentration	Non-contact nuclear density meter with Model: Gammapilot M FMG60 Transmitter: FMG60-N1A1J3D1A Isotope Caesium 137: FSG60-AKA1+Z1 Source Container: FQG61-ACC1AKA1A25A+WAZ1
Mud Pressure	Pressure Transmitter for tailing concentrate Manufacturer: Endress & Hauser Model: Cerabar S PMP71
Rake speed	Internal data from thickener system
Flocculant flow rate	Internal data from flocculant addition system
Mud Level	Level Transmitter for mud level Manufacturer: Endress & Hauser Model: Micropilot FMR62
Underflow rate	Same with feed flow rate
Underflow concentration	Same with feed concentration

**Table 5:** A sample of deep cone thickener processing dataset.

Timestamp	Feed Flow Rate	Feed Concentration	Mud Pressure	Rake Speed	Flocculant Flow Rate	Mud Level	Underflow Rate	Underflow Concentration
9 May 2018 10:20	164.47	16.47	18.41	500.58	4.30	7.01	58.96	59.72
9 May 2018 10:21	169.21	15.51	17.99	500.16	4.06	6.95	61.56	58.88
9 May 2018 10:22	141.78	15.30	16.41	500.56	4.06	6.94	59.97	59.26
9 May 2018 10:23	305.67	25.31	16.11	500.99	4.07	6.97	59.46	58.77
9 May 2018 10:24	328.70	28.28	16.43	501.42	4.43	6.93	59.68	59.43
9 May 2018 10:25	323.96	25.90	17.11	501.56	4.40	6.91	61.40	60.09

**Table 6:** Time series prediction results over our Dataset (best performance displayed in boldface). The size of encoder hidden states m and decoder hidden states p are set as m = p = 64 and 128.

Modles	Enhancement	MAE	RMSE	MAPE	RMLSE
LightGBM	*√*	0.83	1.26	1.27	0.020
RNN(64)	*√*	0.86 ± 0.06	1.28 ± 0.05	1.34 ± 0.09	0.020 ± 0.0008
RNN(128)	*√*	0.78 ± 0.03	1.22 ± 0.02	1.23 ± 0.03	0.019 ± 0.0005
LSTM(64)	*√*	0.81 ± 0.04	1.24 ± 0.04	1.27 ± 0.06	0.019 ± 0.0005
LSTM(128)	×	0.79 ± 0.02	1.22 ± 0.03	1.23 ± 0.04	0.019 ± 0.0004
LSTM(128)	*√*	0.75 ± 0.02	1.19 ± 0.02	1.18 ± 0.03	0.018 ± 0.0003
DARNN(64)	*√*	0.65 ± 0.04	1.02 ± 0.04	1.01± 0.04	0.016 ± 0.0007
DARNN(128)	×	0.64 ± 0.04	1.01 ± 0.04	1.00 ± 0.05	0.016 ± 0.0007
DARNN(128)	*√*	0.61±0.03	1.01±0.03	0.97±0.06	0.016±0.0006

**Table 7:** Time series prediction results of no attention, the spatial attention, the temporal attention, and dual stage attention (best performance displayed in boldface).The size of encoder hidden states m and decoder hidden states p are set as m = p = 128.

Model	Spatial Attention	Temporal Attention	MAE	RMSE	MAPE	RMLSE
DARNN	×	×	0.69 ± 0.05	1.10 ± 0.05	1.12 ± 0.004	0.019 ± 0.0005
	×	*√*	0.64 ± 0.04	1.01 ± 0.04	1.00 ± 0.05	0.016 ± 0.0006
	*√*	×	0.66 ± 0.03	1.02 ± 0.02	1.01 ± 0.04	0.017 ± 0.0007
	*√*	*√*	0.61±0.03	1.01±0.03	0.97±0.06	0.016±0.0006

## Abbreviations

The following abbreviations are used in this manuscript:

AR	Autoregressive integrated
ARMA	Autoregressive integrated moving average
RNN	Recurrent neural network
LSTM	Long short term memory
DARNN	Duel attention recurrent neural network
DCS	Distributed Control System
